# Nanoporous Titanium (Oxy)nitride Films as Broadband Solar Absorbers

**DOI:** 10.1021/acsami.2c01185

**Published:** 2022-04-18

**Authors:** Beatrice R. Bricchi, Luca Mascaretti, Simona Garattoni, Matteo Mazza, Matteo Ghidelli, Alberto Naldoni, Andrea Li Bassi

**Affiliations:** † Micro- and Nanostructured Materials Laboratory, Department of Energy, Politecnico di Milano, Via Ponzio 34/3, 20133 Milano, Italy; ‡ Czech Advanced Technology and Research Institute, Regional Centre of Advanced Technologies and Materials, Palacký University Olomouc, Šlechtitelů 27, 77900 Olomouc, Czech Republic; § Laboratoire des Sciences des Procédés et des Matériaux (LSPM), CNRS, Université Sorbonne Paris Nord, 93430 Villetaneuse, France; ∥ Center for Nano Science and Technology—IIT@PoliMi, Via Giovanni Pascoli 70/3, 20133 Milano, Italy

**Keywords:** broadband solar absorption, pulsed laser deposition, titanium oxynitride, hierarchical nanostructures, solar−thermal conversion

## Abstract

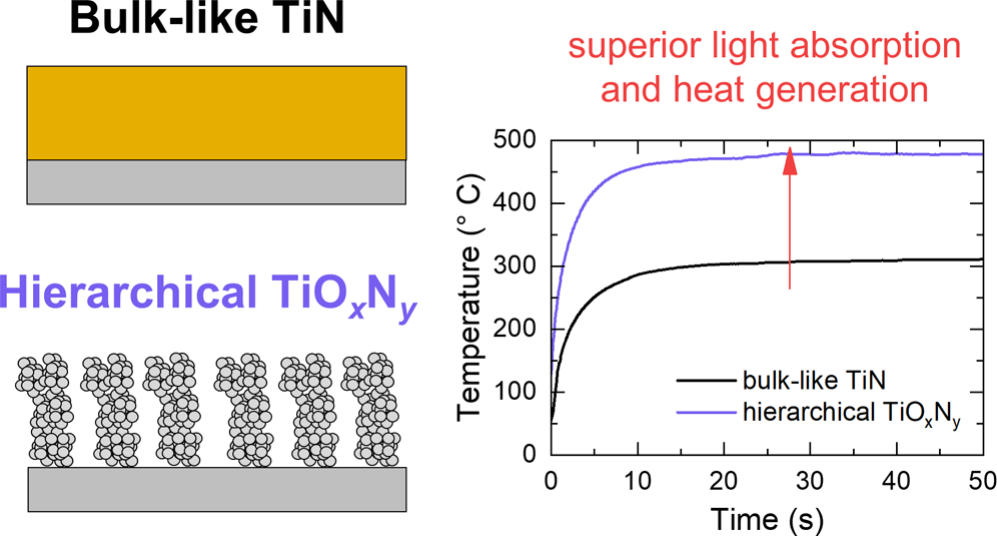

Broadband absorption of solar light is a key aspect in many applications that involve an efficient conversion of solar energy to heat. Titanium nitride (TiN)-based materials, in the form of periodic arrays of nanostructures or multilayers, can promote significant heat generation upon illumination thanks to their efficient light absorption and refractory character. In this work, pulsed laser deposition was chosen as a synthesis technique to shift metallic bulk-like TiN to nanoparticle-assembled hierarchical oxynitride (TiO_*x*_N_*y*_) films by increasing the background gas deposition pressure. The nanoporous hierarchical films exhibit a tree-like morphology, a strong broadband solar absorption (∼90% from the UV to the near-infrared range), and could generate temperatures of ∼475 °C under moderate light concentration (17 Suns). The high heat generation achieved by treelike films is ascribed to their porous morphology, nanocrystalline structure, and oxynitride composition, which overall contribute to a superior light trapping and dissipation to heat. These properties pave the way for the implementation of such films as solar absorber structures.

## 1 Introduction

The abundant and widespread availability of solar energy makes it a potential source for various sustainable energy conversion technologies. Solar–thermal conversion, for example, aims at transforming photons coming from the Sun into heat that could be used for residential, commercial, or industrial applications.^1^ An ideal solar absorber material should exhibit a near-unity and omnidirectional absorption in the 250–2500 nm range of the electromagnetic spectrum, which can be achieved, for example, by metal–insulator–metal (MIM) multilayers.^2,3^ Additionally, broadband absorbers must convert light energy to heat without undergoing thermal degradation, which typically requires the use of refractory metals and oxides^2,4^ or carbon-based components.^5^

Titanium nitride (TiN) is a well-known refractory material employed in the complementary metal oxide semiconductor (CMOS) technology.^6^ TiN in the form of nanoparticles (NPs) or nanostructures exhibits similar plasmonic properties to Au, but it offers several advantages compared to the latter, including the compatibility with semiconductor technology,^6^ high thermal stability,^7,8^ and tunability of its plasmonic resonance by controlling its stoichiometry or crystalline quality.^6^ A further advantage of TiN is that its absorption peak can be extended toward the near-infrared (NIR) range and its optical losses can lead to higher photothermal heating compared to Au.^9,10^ Therefore, it is not surprising that this material has been considered in thermoplasmonics,^11,12^ in which light-to-heat conversion mediated by plasmonic structures is exploited for a wide range of applications,^13^ including solar steam generation,^14^ optical trapping,^15^ and photothermal catalysis.^16,17^

Broadband solar absorbers based on TiN can be realized by coupling multiple TiN nanostructures in different arrangements. For example, ordered arrays or periodic metamaterials made of nm-sized units, such as nanocavities,^16,18^ nanotubes,^19^ and hollow squares,^7^ as well as nonperiodic structures, such as nanodonuts^20^ and nanopillars,^21^ have been reported. These approaches can prevent thermal stresses in MIM multilayers due to the different thermal expansion coefficients in the materials. Apart from optimizing the thermal stability of the solar absorber, the achievement of complete absorption with a simple fabrication method is also challenging.^21^ A simple approach in this regard is represented by porous films made of NP assemblies grown on a flat substrate, in which broadband absorption can be achieved as a result of coupling among individual localized surface plasmon resonances (LSPRs) of the NPs and light trapping effects (i.e., multiple reflections/scattering) promoted by the nm-scale porosity.^22,23^ Moreover, the poor thermal conductivity of NPs compared to bulk materials^24^ may limit the thermal transfer to the substrate material, thus giving rise to higher surface temperatures upon light absorption. An alternative approach to realize broadband solar absorbers consists of coating porous anodic alumina templates with a thin metallic layer.^25,26^ Apart from nanostructuring, broadband absorption can also be achieved by oxidation of TiN or nitridation of TiO_2_, thus realizing titanium oxynitrides (TiO_*x*_N_*y*_). By properly tuning the film stoichiometry, the so-called double-epsilon-near-zero (2ENZ) behavior in the dielectric function can be achieved, which can lead to multiple plasmonic resonances.^27,28^

Compared to standard NP-assembled materials, TiN thin films can be synthesized by physical/chemical vapor deposition methods including magnetron sputtering,^29−31^ glancing angle deposition,^32,33^ atomic layer deposition (ALD),^34,35^ and pulsed laser deposition (PLD).^36,37^ The latter, in particular, allows depositing virtually any material with high tunability of morphology and structure: compact layers,^36,38^ surface-supported NPs,^39,40^ hierarchical tree-like films,^37,41−43^ and ultraporous foams^44,45^ can be obtained by controlling the background gas pressure and/or the target-to-substrate distance at room temperature. The latter is an important feature in terms of process energy utilization and usage of flexible substrates. In this work, we exploit the versatility of PLD to deposit TiN/TiO_*x*_N_*y*_ nanostructured films in a controlled atmosphere (from vacuum up to 100 Pa of N_2_/H_2_) to tune the morphology, structure and optical absorption, with the aim of realizing broadband solar absorbers. Tree-like films deposited at 50 and 100 Pa, in particular, exhibited the highest optical absorption over the whole ultraviolet–visible–near-infrared (UV–visible–NIR) range. All of the investigated films were tested by noncontact thermal measurements under moderate solar irradiation. We show that the TiO_*x*_N_*y*_ film deposited at 100 Pa produced the highest temperature of ∼475 °C under 17 Suns, which was ascribed to its broadband optical absorption arising both from the oxynitride composition and porous morphology. Our results open the way to the utilization of TiN-based broadband solar absorbers with controlled functional properties fabricated by PLD in solar–thermal devices.

## 2 Experimental Methods

### 2.1 Sample Preparation

PLD was performed in a vacuum chamber equipped with mass flow controllers to tune the partial gas pressure. Ablation was performed with a ns-pulsed laser (Nd:YAG, second harmonic, λ = 532 nm) with pulse duration in the 5–7 ns range and repetition rate of 10 Hz. The laser pulses were focused on the target through a viewport with a fluence of 6.5 J cm^–2^ (incidence angle of 45°, laser energy of 420 mJ pulse^–1^, and elliptical laser spot with 6 mm^2^ area). The target material was stoichiometric TiN (99.9% purity, Mateck Gmbh), which was mounted on a roto-translational manipulator ensuring a uniform ablation. After evacuating the chamber to the base vacuum of 3 × 10^–3^ Pa, depositions were performed at room temperature still in vacuum or N_2_/H_2_ (95/5%, 5.0 purity) background gas mixture at the overall pressure equal to 10, 20, 50, and 100 Pa. Si(100), soda-lime glass, and Ti plate substrates were cleaned in an ultrasonic bath with isopropanol and mounted on a rotating sample holder placed head-on the target at a fixed distance of 50 mm. The deposition time was set at 2 h.

### 2.2 Material Characterization

The thin film morphology was evaluated by means of a field emission scanning electron microscope (SEM, Zeiss SUPRA 40) on samples grown on silicon. The SEM microscope is equipped with an Oxford Instruments Si(Li) detector for energy-dispersive X-ray spectroscopy (EDX), which was employed to qualitatively estimate the atomic percentage (atom %) of Ti, N, and O in the films, by employing an accelerating voltage of 10 kV. The quantitative chemical composition was characterized by X-ray photoelectron spectroscopy (XPS) with a PHI 5000 VersaProbe II XPS system (Physical Electronics) with a monochromatized Al Kα source (15 kV, 50 W) and a photon energy of 1486.7 eV. Depth profiling was performed by Ar^+^ sputtering with 2 kV beam energy (2 × 2 mm^2^ area). The chemical composition of selected films was further analyzed by EDX mapping in a high-resolution transmission electron microscope (HRTEM, Titan G2) operated in scanning TEM mode (STEM) using a Super-X system with four silicon drift detectors (Bruker). STEM images were taken with a high-angle annular dark-field imaging (HAADF) detector (Fischione, model 3000). TEM lamellae were prepared with a FEI Helios Focused Ion Beam/SEM (Thermo Scientific). The crystalline structure of the films was investigated by X-ray diffraction (XRD) using a high-resolution X-ray powder diffractometer (PANalytical X’Pert Pro MPD) with Co Kα radiation (λ = 0.1789 nm). The measurements were performed in Bragg–Brentano geometry in a 2θ range of 22–100° with a step size of 0.033°. Further qualitative information about stoichiometry/composition of the films was gained by Raman spectroscopy using a Renishaw InVia micro-Raman spectrometer equipped with a diode-pumped solid-state laser (λ = 660 nm, incident power on the sample of 0.94 mW, spectral resolution ∼3 cm^–1^). The optical characterization of the films in the spectral range 250–2000 nm was evaluated by transmittance (*T*) and reflectance (*R*) spectra on samples deposited on soda-lime glass using a PerkinElmer Lambda 1050 spectrophotometer equipped with an integrating sphere (150 mm diameter). In the spectral range 1330–25 000 nm, transmittance and reflectance spectra on samples deposited on silicon were acquired by a vacuum Fourier transform infrared (FTIR) Vertex 80v spectrophotometer. The optical properties of samples grown on Si substrates were further investigated by spectroscopic ellipsometry (J. A. Woollam) in the range of 0.6–6.5 eV (0.1 eV energy interval, 65 and 75° angles of incidence).

### 2.3 Infrared Thermal Imaging

The heat generation produced by the films under irradiation was evaluated by measuring the temperature of the Ti substrate (thickness 0.125 mm) by an FLIR X6580sc infrared camera. The thermal camera was placed on the back side of the samples that were irradiated from the front side with a 1000 W solar simulator (Sciencetech A4 Lightline C250) equipped with an AM 1.5G filter and an aspheric condenser lens (ACL25416U, Thorlabs). The samples were kept in a custom-made vacuum cell in Ar at atmospheric pressure (upon purging the air in the chamber with an Ar flow for 10 min) equipped with a sapphire viewport on the front side (420GSG040-saphir, Pfeiffer Vacuum GmbH) and a CaF_2_ viewport (VPCH512, Thorlabs) on the back side. The temperatures measured by the thermal camera were corrected by the spectral emissivity of the Ti plates measured by FTIR spectroscopy at room temperature and averaged in the sensitivity range of the thermal camera (2500–5500 nm; see the Supporting Information for additional details).

## 3 Results and Discussion

**Figure 1:**
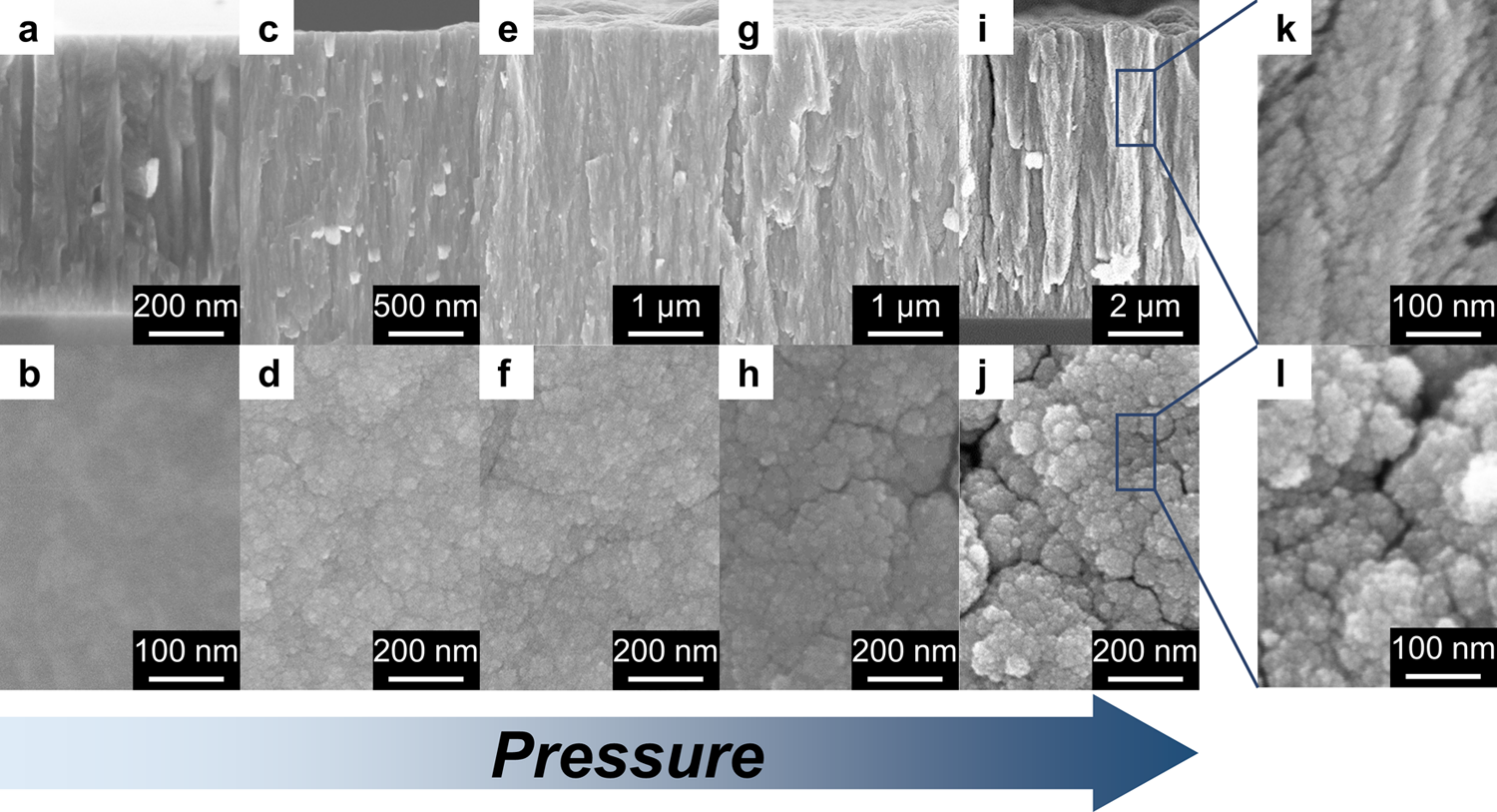
SEM cross-sectional (first row) and top views (second row) images of the films deposited (a, b) in vacuum, (c, d) at 10 Pa, (e, f) at 20 Pa, (g, h) at 50 Pa, and (i, j) at 100 Pa of N_2_/H_2_; (k) cross-sectional and (l) top-view magnifications of the film deposited at 100 Pa.

TiN and TiO_*x*_N_*y*_ films with different morphologies were synthesized by varying the background pressure during the deposition from vacuum up to 100 Pa of N_2_/H_2_ to favor Ti–N bonds formation during film growth. The SEM cross section and top-view micrographs are reported in Figure 1. The film deposited in vacuum shows a compact and dense columnar structure (Figure 1a) and a smooth surface (Figure 1b). For the samples deposited at 10 and 20 Pa (Figure 1c–f), the columns composing the film exhibit a slight deviation from a perfectly vertical growth direction with a consequent increase of porosity. A further increase of the background pressure during the deposition leads to the growth of a hierarchical nanoparticle assembly structure (Figure 1g,h), clearly visible for the film deposited at 100 Pa (Figure 1i–l). As a consequence of the increase of film porosity with background pressure during deposition, the thickness of the films for a deposition duration of 2 h increased from 2.4 μm for the vacuum-deposited film up to 3, 4.5, 6, and 9.5 μm for the ones deposited at 10, 20, 50, and 100 Pa, respectively.

A morphological transition from compact to nanoporous films by increasing the background gas pressure is typical of the PLD process.^41,46^ Upon the interaction of the focused pulsed laser on the target material in a controlled atmosphere, i.e., laser ablation, a plasma plume is generated and expands from the target surface toward the substrate. In low-pressure conditions, the ablated species possess high kinetic energy and lead to a compact or bulk-like growth. In high-pressure conditions, the background gas molecules and the ablated species undergo collisions, which decrease the kinetic energy of the latter and lead to a cluster-assembled growth regime. At very high background pressures, foam-like films can be achieved.^44,45^ The hierarchical nanoparticle assembly (tree-like) morphology observed for the films deposited at 50 and 100 Pa (Figure 1g–j) has been frequently observed for various oxides^41−43,47^ as well as for TiN.^37^ Assuming the same density as bulk TiN (5.24 g cm^–3^) for the film deposited in vacuum, densities ranging from ∼3 to ∼1 g cm^–3^ by increasing the deposition pressure from 10 to 100 Pa could be estimated by quartz microbalance measurements, corresponding to surface areas up to ∼100 m^2^ g^–1^.^37^

**Figure 2:**
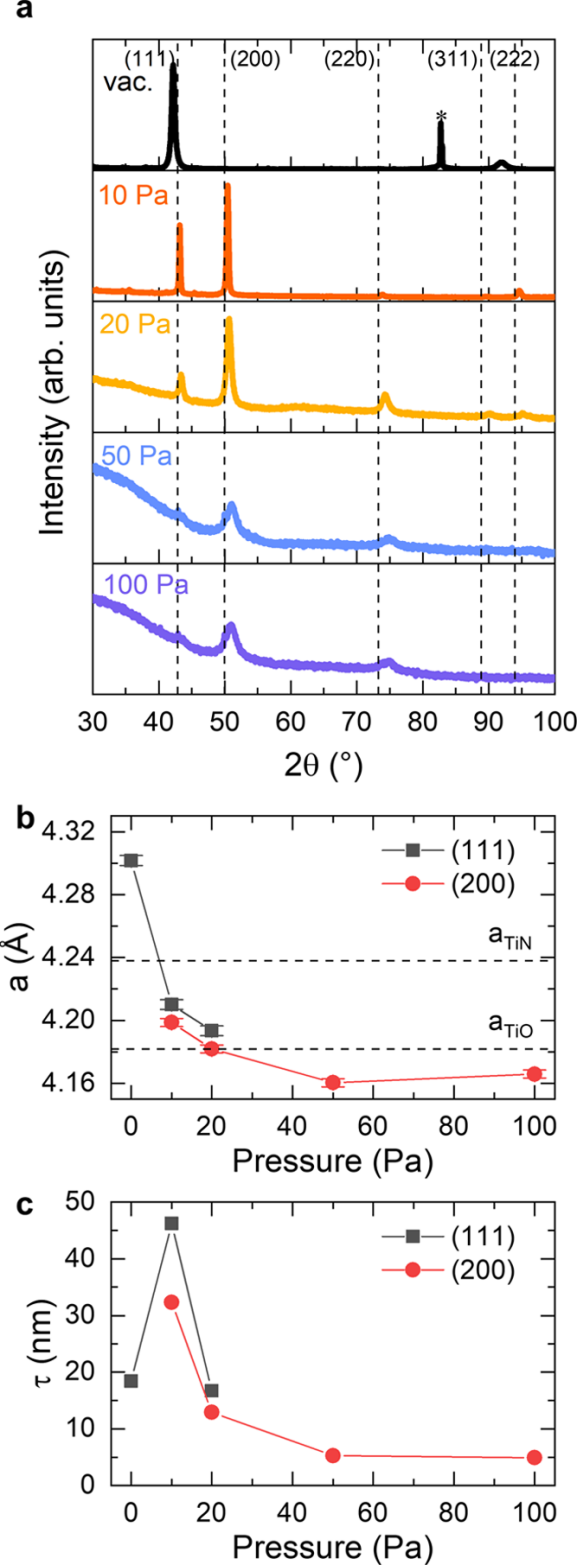
(a) XRD patterns of TiN films deposited in vacuum on Si substrate and at 10, 20, 50, and 100 Pa N_2_/H_2_ on glass substrates (asterisk: Si substrate; vertical dashed lines: reference TiN peaks). (b) Lattice constant *a* and (c) average domain size τ evaluated for the (111) and (200) XRD reflections. The horizontal dashed lines in (b) correspond to the lattice constants for bulk TiN (4.238 Å) and γ-TiO (*a*_TiO_ = 4.182 Å). Reference data for TiN taken from PDF database card no. 01-087-0633 and for TiO from ref (55).

The structural properties were investigated by XRD in Bragg–Brentano geometry, which is sensitive to the preferential orientation of crystalline domains along the film growth direction. The diffractograms for all of the films exhibited the characteristic peaks of the cubic phase of TiN (**Fm**3̅*m* space group), but a remarkable change of texture and shift of the diffraction angles were found by increasing the background pressure during deposition. The vacuum-deposited film exhibited the (111) and (222) reflections at lower diffraction angles than the corresponding counterparts in bulk TiN. On the contrary, the film deposited at 10 Pa exhibited also the (200), which was the most intense in this case, (220) and (311) reflections (the latter slightly visible), all of them at higher diffraction angles than in the case of bulk TiN. By further increasing the deposition background pressure all of the peaks decreased in intensity and shifted to higher angles, while an amorphous background emerged at *P* > 20 Pa (Figure 2a). The peak shift effect can be better highlighted by evaluating the lattice constant *a* using the Bragg’s law for cubic crystal system from the (111) reflections (vacuum, 10 and 20 Pa) and (200) reflections (10–100 Pa), as shown in Figure 2b. Taking as reference value for bulk TiN *a*_TiN_ = 4.2380 Å, the vacuum-deposited film showed *a* > *a*_TiN_. For all of the other films, conversely, *a* < *a*_TiN_ and the lattice constant decreased with the deposition pressure. Interestingly, for the films deposited at *P* > 20 Pa the lattice constant was lower than that of cubic titanium monoxide, γ-TiO (*a*_TiO_ = 4.182 Å), which has the same rock-salt crystal structure as TiN (this comparison is introduced because of the presence of oxygen in the films, see below). Furthermore, the average domain size (τ) along the film growth direction was evaluated through the broadening of the diffraction peaks using the Scherrer equation on the (111) reflection for the films deposited in vacuum and 10–20 Pa, and on the (200) reflection for films deposited at 10–100 Pa (Figure 2c). For the film deposited in vacuum, τ ∼ 18 nm; this value increases reaching a maximum for the film deposited at 10 Pa (τ ∼ 46 nm for the (111) reflection and τ ∼ 32 nm for (200) reflection), and finally decreases with the background pressure down to τ ∼ 5 nm for both the films deposited at 50 and 100 Pa.

XRD data could be interpreted in terms of the growth regimes induced by the increase of deposition background gas pressure. The features of the film deposited in vacuum (*a* > *a*_TiN_) are consistent with two effects: on the one hand, a nitrogen sub-stoichiometry induced by non-stoichiometric transfer from the target and, therefore, nitrogen loss;^48,49^ on the other hand, in-plane compressive stresses due to highly energetic particles having a peening effect on the growing film.^29^ The residual stresses indeed were retrieved by evaluating the macro-strain as ε_macro_ = |*a*–*a*_ref_|/*a*_ref_ = 0.015, that is consistent with a compact film with compressive stress deposited by physical vapor deposition.^31,50^ By ablating the target in the presence of a N_2_/H_2_ background gas, instead, the species in the plume slowed down because of the collisions with the gas molecules. As a consequence, at 10 Pa a trade-off between in-plume cluster nucleation and sufficiently large kinetic energy of the ablated species probably promoted a good crystallization with small residual stresses (ε_macro_ = 0.006). A further pressure increase in the chamber promoted a less directional ablation plume and a low kinetic energy of the clusters formed in the plume. Therefore, a progressive decrease of the average domain size in parallel with amorphization of the film is expected.^41,51^ Another effect coming into play was a partial oxidation of the films due to residual impurities in the chamber (which might be elucidated by plasma diagnostics techniques,^46,52^ that were unavailable for the current study) and air exposure, especially for the porous films. Previous studies, indeed, reported a decrease of the lattice parameter (down to 4.16 Å) as well as of the average domain size (2.8 nm) by decreasing the flow rate of N_2_ and increasing that of O_2_ during magnetron sputtering experiments, thus producing titanium oxynitrides, i.e., TiO_*x*_N_*y*_.^53^ For such materials, a lower lattice parameter compared to both standard TiN and TiO materials could be explained by the presence of ion vacancies.^54^

**Figure 3:**
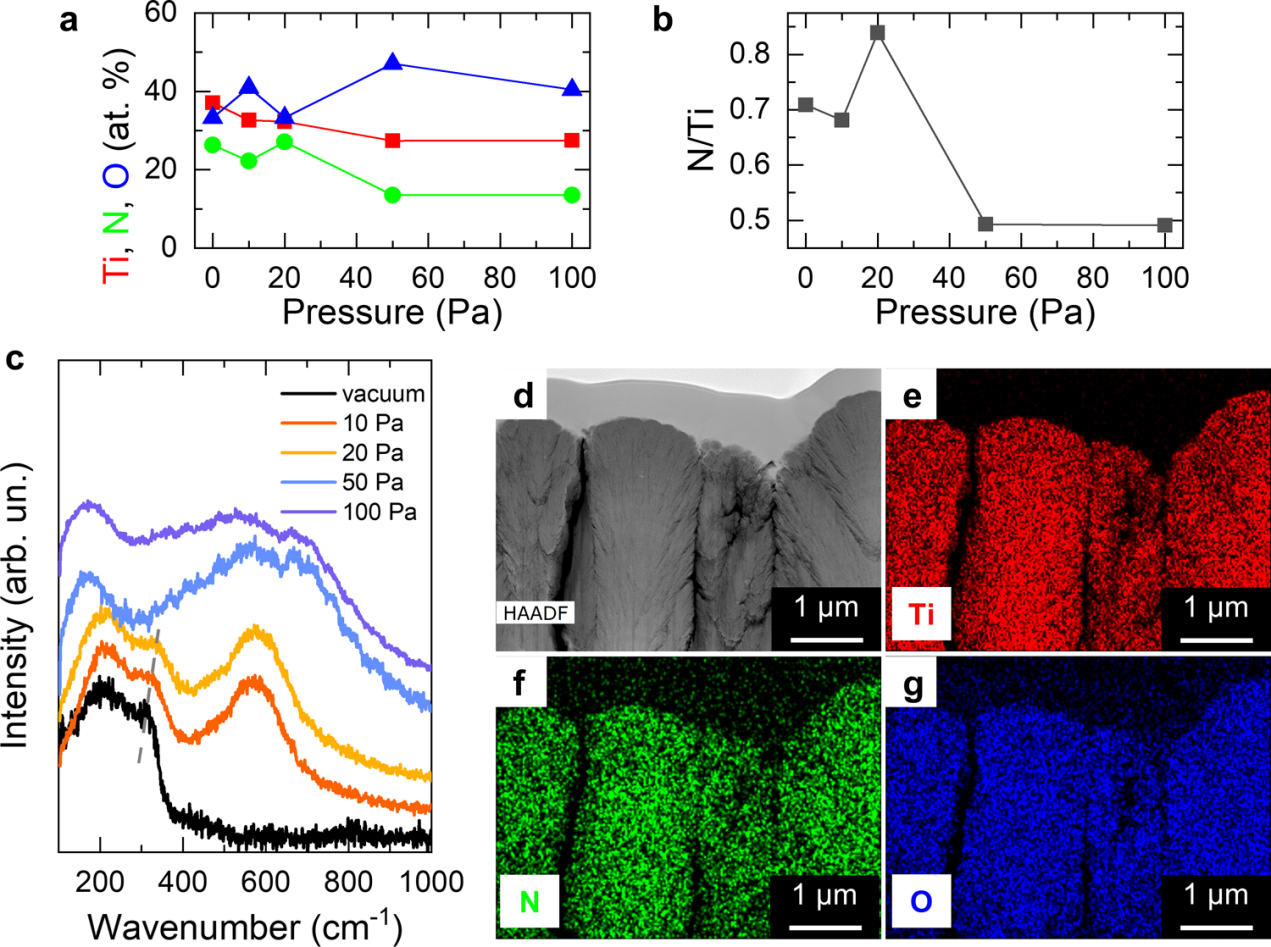
(a) Atomic percentage of Ti, N, and O by XPS depth profiling (60 s sputtering time), (b) N-to-Ti ratio (N/Ti), and (c) Raman spectra of the films deposited in vacuum and at 10–100 Pa. (d) High-angle annular dark-field STEM (HAADF-STEM) image and corresponding EDX mapping of Ti (e), N (f), and O (g) for the film deposited at 100 Pa.

The data presented in Figures 1 and 2 show the key role of the N_2_/H_2_ deposition pressure in controlling not only the morphology but also the structure and the composition of the films. Since the latter itself deeply affects the functional properties of titanium nitride-based materials, a further compositional characterization was addressed by different techniques (Figure 3). EDX microanalysis was carried out to qualitatively estimate the atomic content (atom %) of Ti, N, and O in the films and to evaluate the nitrogen to titanium (N/Ti) ratio. Preliminary measurements on the TiN target revealed an apparent under-stoichiometric composition (i.e., N/Ti < 1) with N/Ti = 0.8 and 13 atom % O (Figure S1). A substantial amount of oxygen (>30 atom %) was also found in all of the films (not shown). It is well known that a native thin oxide surface layer usually forms on TiN-based materials upon air exposure.^56,57^ The quantitative compositional analysis was therefore addressed by XPS after 60 s depth profiling to remove the surface oxide layer (Figure 3a,b and Table S1). The Ti content decreased from the maximum exhibited by the film deposited in vacuum (∼37%) by introducing the N_2_/H_2_ gas with 10 and 20 Pa total pressure (∼32% in both cases), and it further decreased at high pressure (∼27% for the films deposited at 50 and 100 Pa). The nitrogen content varied in the range of 22–27% up to the pressure of 20 Pa and it decreased to ∼13% at 50 and 100 Pa. The oxygen content was instead higher than 30% in all of the cases, with the maximum value found for the film deposited at 50 Pa (O ∼ 47%). The most relevant feature of the porous films was their low N/Ti ratio (∼0.5) compared to that of all of the other films (N/Ti ∼ 0.7–0.8). These data suggest partial oxidation of the films not only at their surface but also along their thickness, especially for those grown at 50 and 100 Pa. More information in this regard was gained by Raman spectroscopy (Figure 3c). The vacuum-deposited film exhibited evident acoustic Raman bands (∼200–300 cm^–1^) without optical bands, which was ascribed to nitrogen vacancies and, therefore, under-stoichiometry (i.e., TiN_*x*_ with *x* < 1, see Note S1 for more details on the interpretation of Raman spectra in TiN). The Raman spectra for the films deposited at 10 and 20 Pa exhibited a shift of the acoustic band (from ∼310 cm^–1^ for the film deposited in vacuum to ∼330 cm^–1^ for that deposited at 20 Pa) and the appearance of the optical Raman band (∼500–600 cm^–1^), which is associated to Ti vacancies. By further increasing the deposition pressure, the Raman spectra became broader and exhibited a band at ∼170 cm^–1^. Further analysis was performed by STEM-EDX mapping on lamellae prepared from a film deposited at 100 Pa (Figure 3d–g). The maps show that Ti, N, and O are present all along the tree-like structure. The measured elemental composition was Ti = 43.4 atom %, N = 23.5 atom %, and O = 33.1 atom %, with N/Ti = 0.54 in agreement with XPS data. Therefore, by comparing the data on the chemical composition by XPS (Figure 3a,b) and by STEM-EDX (Figure 3d–g) with the Raman spectra (Figure 3c), it is possible to hypothesize that the film deposited in vacuum consists of under-stoichiometric TiN_*x*_ with a limited degree of oxidation. All of the other films, meanwhile, likely correspond to TiO_*x*_N_*y*_ with a degree of oxidation that increased with the deposition pressure, leading to the observed shifts of Raman bands.^53^ In particular, the films deposited at 50 and 100 Pa likely feature a substantial amount of amorphous TiO_*x*_, which is suggested by the Raman band at ∼170 cm^–1^ (Figure 3c) and by the low N/Ti ratio (∼0.5). This hypothesis was confirmed by high-resolution XPS analysis of the pristine surface of the film deposited at 100 Pa (Figure S2 and Table S2), which revealed that the binding energies of the peaks in the Ti 2p and O 1s regions were comparable to reduced titanium dioxide. The extensive oxidation in the case of the porous films can be understood by considering that their low density and high porosity favor the saturation of ion vacancies by exposure to oxygen. These hypotheses are in agreement with the structural data provided by XRD (Figure 2), which also suggested an increasing amorphization and deviation from TiN_*x*_ to TiO_*x*_N_*y*_ stoichiometry richer in O by increasing the background gas pressure (Figure 2), as well as with previous studies.^37,53,54^

**Figure 4:**
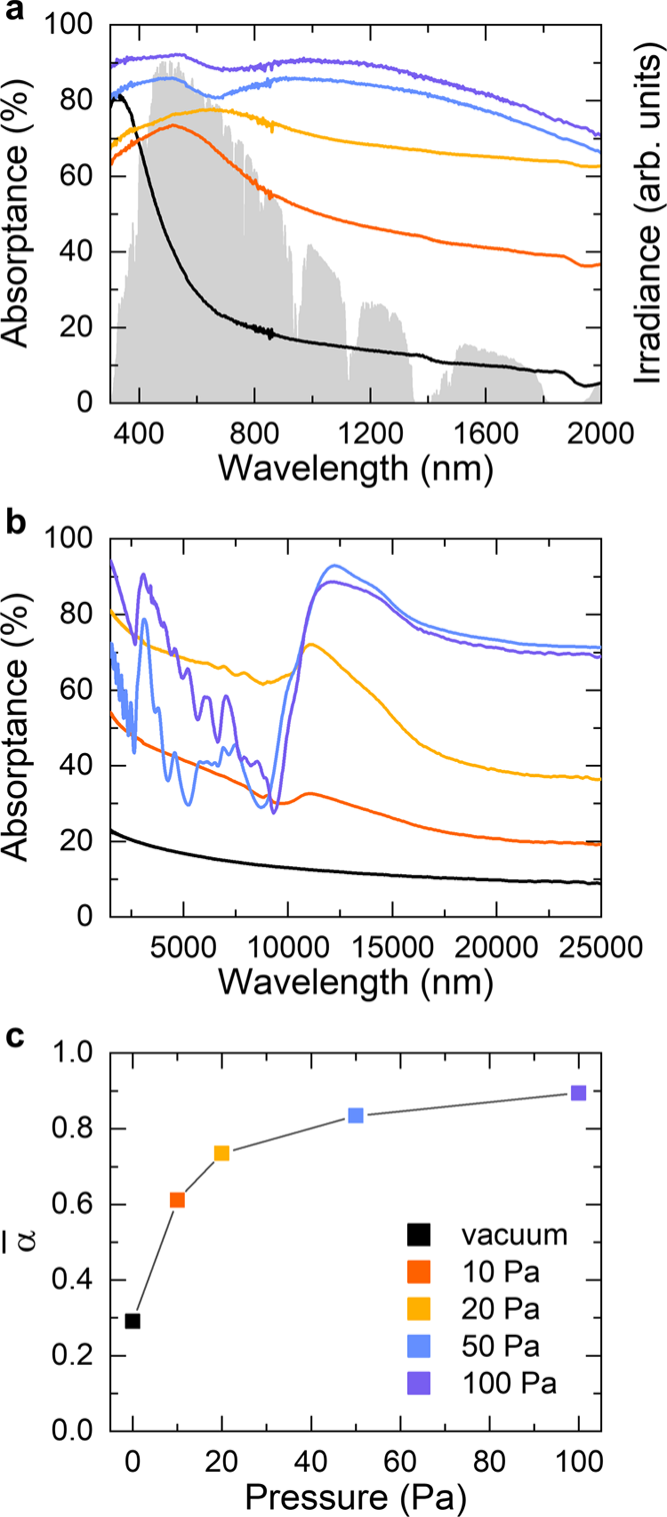
(a) Optical absorptance of the TiN films compared to the spectral solar irradiance (ASTM G173-03 AM 1.5 Global). (b) Optical absorptance in the MIR range retrieved by FTIR spectroscopy. (c) Spectrally averaged absorptance in the 280–2000 nm wavelength range. For all panels, the color legend is reported in (c).

The morphological, structural, and compositional evolution of the films with the increase of deposition pressure discussed above was accompanied by a change in the optical properties (Figure 4). Absorptance spectra were retrieved by transmittance (*T*(λ)) and reflectance (*R*(λ)) measurements (Figure S3) according to the formula *A*(λ) = 1 – *T* (λ) – *R*(λ) (see Note S2 for details on the measurements in the different wavelength ranges and on data treatment). Figure 4a,b shows the optical spectra in the ultraviolet–visible–near-infrared (UV–vis–NIR, i.e., 250–2000 nm) range compared to the standard solar irradiance and in the medium-infrared (MIR, i.e., 1330–25 000 nm) range, respectively. The absorptance monotonically increased and the overall behavior of the films dramatically changed by increasing the deposition pressure. In particular, the film deposited in vacuum showed zero transmittance (Figure S3a,b) and a well-defined reflectance minimum (Figure S3c,d) or absorptance maximum in the UV region of the electromagnetic spectrum (i.e., at 318 nm, Figure 4a). This behavior is very similar to that of bulk TiN, which exhibits a well-defined interband transition threshold at ∼500 nm and a low absorption/high reflectance due to intraband transitions at longer wavelengths.^6^ For the films deposited at 10 and 20 Pa the absorption peak red-shifted to 516 and 658 nm, respectively, and broadened, thus leading to a higher absorptance in the full UV–MIR range than the film deposited in vacuum. Additionally, these films exhibited a non-zero transmittance in the MIR range (maximum ∼10% at ∼10 μm, Figure S3b). Since the absorption maximum (or the reflectance minimum) position is associated with the plasma frequency of the film, its redshift can be explained by a change of charge carrier density, which implies a change of stoichiometry or composition.^27,58^ In this case, according to XPS analysis (Figure 2a,b), a lower titanium content contributed to the redshift of the absorption peak, thus decreasing the metallic character of the films. Moreover, the broadening of the absorption could be associated with a transition from a smooth film surface to a nanostructured one (Figure 1), which can promote light scattering and light trapping phenomena. This effect became more evident for the films deposited at 50 and 100 Pa, which exhibited a broadband absorption in the whole investigated range (Figure 4a,b). These films exhibited a non-zero transmittance at λ > 530 nm (Figure S3a), which increased up to ∼60% at MIR wavelengths, and then abruptly decreased to zero (Figure S3b). As a result, the absorptance exhibited broad maxima in the visible (λ ∼ 500 nm), NIR (λ ∼ 1000 nm), and MIR (λ ∼ 13 000 nm) ranges of the electromagnetic spectrum, with an absolute minimum at ∼10 000 nm. Various effects could explain such an optical behavior. The main contribution is likely due to oxygen incorporation in the films, thus featuring a TiO_*x*_N_*y*_ composition, which is supported by XPS, Raman, and EDX-STEM data (Figures 3 and S2). TiO_*x*_N_*y*_ materials show indeed a non-zero transmittance^53,59^ and a more extended energy range for interband transitions involving the additional O 2p orbitals.^60^ On the other hand, broadband absorption was also shown for highly substoichiometric TiO_*x*_^22^ and commercial TiN^23^ NPs assemblies and for TiN nanopillars.^21^ In all of these cases, a superposition of individual localized surface plasmon resonances (LSPRs) of the individual units, i.e., plasmon hybridization,^61,62^ increased and broadened the overall absorption of the film. Yan et al. further showed that the porous nanostructure promoted light trapping due to multiple reflections and scattering of light as well as a reduced reflectance at the air–solid interface due to an effective graded refractive index layer.^22^ Similarly, in the present case, an increase of light scattering ability of the films with the morphological transition from compact to porous was assessed by retrieving the haze factor, i.e., the ratio between the diffuse and total components of the transmittance (Note S2), which increased with the deposition pressure for the films deposited at 50 and 100 Pa (Figure S3e). Additional information was gained by spectroscopic ellipsometry measurements on all of the films and by extracting the pseudo-dielectric constants by direct inversion^63^ (Figure S4 and Note S3). Only the film deposited in vacuum showed a strongly metallic behavior with ⟨ε_1_⟩ going from positive to negative (Figure S4a) and ⟨ε_2_⟩ increasing in the near-infrared range (Figure S4b), while ⟨ε_1_⟩ was positive for all of the other films, similarly to a TiN/TiO_2_ intermixed material modeled by effective medium approximation theory (Figure S4a), as found in previous works.^27^ The pseudo-dielectric constant therefore reproduced an effective behavior of the material arising from the properties of individual nanostructures and scattering or light trapping effects related to nanoparticle assembly. These insights further confirmed the hypothesis made from the structural and chemical characterization (Figures 2 and 3). To conclude, a TiN_*x*_ (with *x* < 1) stoichiometry was assigned to the film deposited in vacuum and a TiO_*x*_N_*y*_ stoichiometry to all of the other films, with a higher (amorphous) oxide fraction for the porous films (deposited at 50 and 100 Pa).

To evaluate the overall performance of the films as solar absorbers, the spectrally averaged solar absorptance was calculated according to the formula^64^
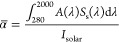
1where *S*_s_(λ) is the spectral solar irradiance (AM 1.5G), *I*_solar_ is the total irradiance, and the calculation is performed in the investigated wavelengths range, i.e., 280–2000 nm. Figure 4c shows that this quantity monotonically increased with the deposition pressure. In particular, α̅ abruptly increased from ∼0.29 for the film prepared in vacuum to ∼0.61 for the film deposited at 10 Pa, and then it reached the maximum value of ∼0.89 at the deposition pressure of 100 Pa, thus confirming the trend discussed above. The α̅ value found for the film deposited at 100 Pa is comparable to other solar absorbers reported in the literature, such as a two-dimensional (2D) Ta photonic crystal (α̅ = 0.864),^64^ a Ti/Al_2_O_3_/Ta plasmonic metamaterial (α̅ = 0.913),^65^ TiN nanopillars (α̅ = 0.94),^21^ and TiN/TiN NPs/SiO_2_ ceramic layer (α̅ = 0.95).^23^ Hence, the data presented in Figure 4 highlight the possibility of achieving a broadband solar absorber behavior for the tree-like TiO_*x*_N_*y*_ films deposited at high pressures.

**Figure 5:**
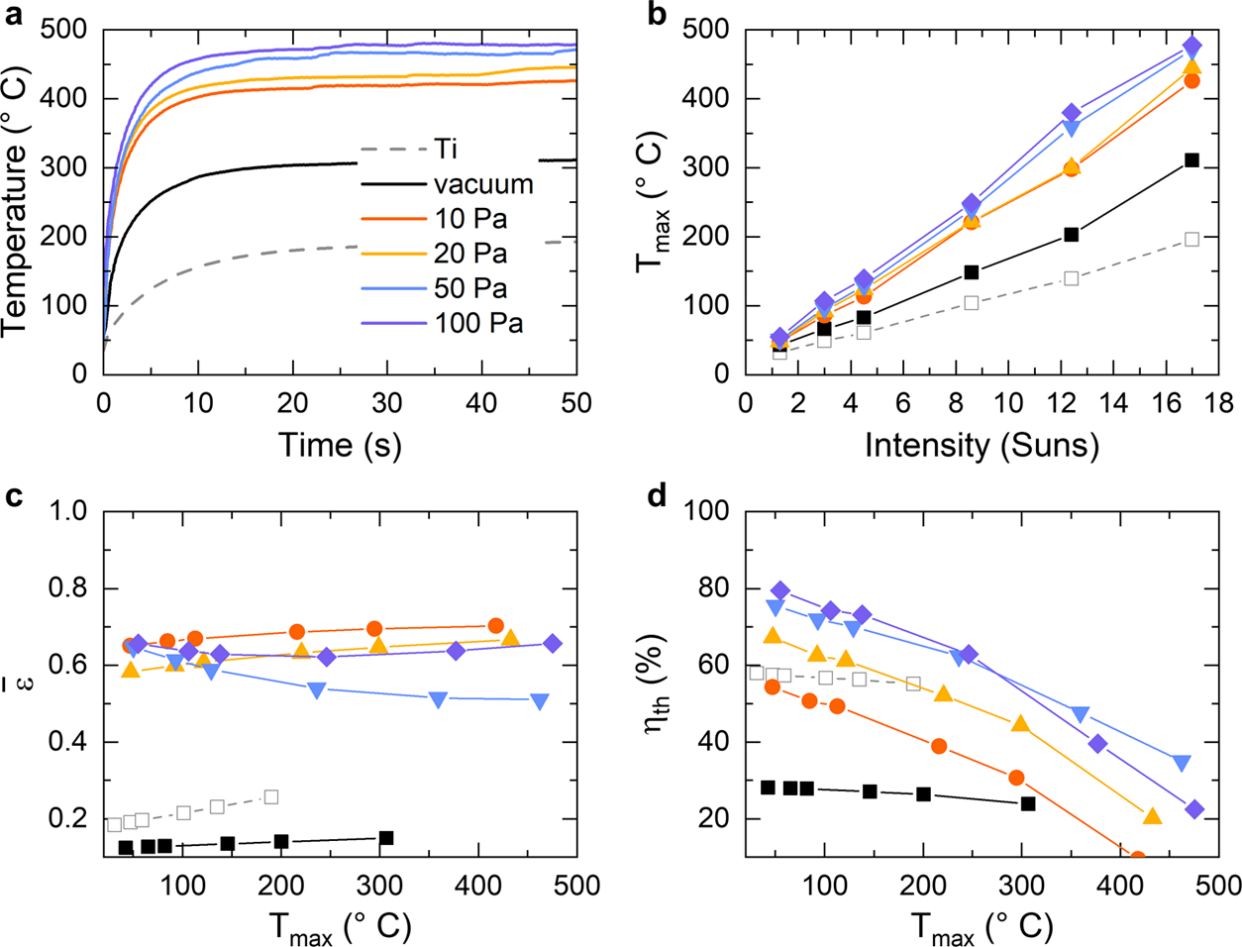
Solar–thermal performance of the investigated films. (a) Temperature profiles under 17 Suns as a function of time. (b) Maximum temperature (steady-state value at the end of each experiment) measured as a function of the solar power (1.3–17 Suns). (c) Spectrally averaged emittance and (d) thermal transfer efficiency as a function of the maximum temperature under irradiation. All panels report also the data for an uncoated titanium substrate.

The performance of the TiN/TiN_*x*_O_*y*_ films for solar–thermal conversion applications was evaluated by non-contact thermal measurements under solar irradiation (Figure 5). The temperature reached under solar-simulated light from 1.3 to 17 Sun (1 Sun = 100 mW cm^–2^) was measured with an infrared thermal camera pointing the back surface of the films deposited on titanium substrates (see Figure S5 for the experimental details and Figure S6 for a discussion on the substrate effect). The samples were kept in a homemade vacuum cell under an inert Ar atmosphere to prevent surface oxidation and to allow multiple experiments under different irradiation conditions for extended periods of time.^16,66,67^Figure 5a shows the temperature profiles during time under the maximum irradiation conditions (i.e., 17 Suns) for all of the investigated films compared with an uncoated Ti substrate (see Figure S7 for the results for all of the films under all irradiation conditions). It is possible to observe that the films heated up very quickly (i.e., in less than 10 s), contrarily to the bare Ti plate, and they reached a steady-state temperature after ∼20 s. The steady-state temperature value measured at the end of the experiment, hereinafter labeled as *T*_max_ (maximum temperature) is shown for all of the films under all of the investigated solar intensities in Figure 5b. The generated temperature increased with the deposition pressure up to the maximum value of ∼475 °C under 17 Suns irradiation for the film deposited at 100 Pa, which is a result comparable to the temperature generated by periodic TiN cylindrical nanocavities under the same moderately concentrated solar condition.^16^ This outcome is even more interesting considering that similar temperatures were reached by metallic nanoparticles array and TiN periodic nanostructures (i.e., nanotubes and trench) under laser irradiation with 10^6^-fold and 10^4^-fold greater power densities, respectively.^19,68^ Notably, the films did not undergo any degradation upon solar irradiation. Raman spectroscopy experiments (Figure S8), indeed, revealed only minor changes in the spectra compared to the pristine samples. While the experiments shown in Figure 5a,b were carried out in an inert atmosphere, scale-up in environmental conditions could be realized by including a capping layer on top of the films, i.e., Al_2_O_3_ or Si_3_N_4_ by ALD.^66,67^

To characterize the solar–thermal performance of the films, the spectrally averaged emissivity at each irradiation condition in correspondence of *T*_max_ was calculated as^64^
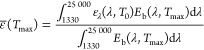
2

In eq 2, all of the quantities are referred to the NIR–MIR range (i.e., the range investigated by FTIR measurements) because it is the typical range for thermal emission corresponding to surface temperatures of a few hundreds of °C. ε_λ_ (λ,*T*_0_) = *A*_FTIR_ (λ,*T*_0_) is the spectral emissivity according to the Kirchhoff’s law of thermal radiation evaluated at room temperature (see a related discussion on the systematic errors introduced by instrumental limitations of FTIR spectroscopy in Note S2), while *E*_b_ (λ, *T*_max_) is the black-body irradiance given by Planck’s law,
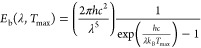
3where *h* is the Planck constant, *c* is the speed of light, and *k*_B_ is the Boltzmann constant. Figure 5c shows the values for ε̅ (*T*_max_) for all films under all irradiation conditions compared to the uncoated Ti substrate. The film deposited in vacuum exhibited a very low emittance (0.12–0.14) due to its highly metallic and reflective properties (Figure S3d). For all of the other films, the emittance values fell in the interval 0.5–0.7, with the minimum ε̅ (*T*_max_) = 0.51 found for the film deposited at 50 Pa. The moderate difference in emittance values for the films deposited at 10–100 Pa (Figure 5c) compared to the dramatic increase of the spectrally averaged absorptance with the deposition pressure (Figure 4c) is due to the transparency window in the NIR range (up to ∼10 μm) for the porous films (Figure S3b).

Taking into account the values of α̅ (Figure 4c) and ε̅ (*T*_max_) (Figure 5c), the thermal transfer efficiency was evaluated as^1^
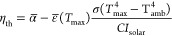
4where σ = 5.670367 × 10^–8^ W m^–2^ K^–4^ is the Stefan–Boltzmann constant, *T*_amb_ = 25 °C is the ambient temperature during the experiments, and *C* is the solar concentration factor (1.3–17). Figure 5d shows that the samples deposited at pressures ≥20 Pa outperformed the bare Ti substrate. The maximum thermal transfer efficiency was found for the film deposited at 100 Pa at 1.3 Sun (η_th_ ∼ 79% at *T*_max_ ∼ 54.9 °C), and the efficiency values decreased with the irradiation intensity for all of the investigated films. This is because the radiative losses are very limited at room temperature, but they follow a ∼*T*^4^ dependence, thus making the emittance contribution increasingly more relevant at increasing temperatures. It is possible to note that under 17 Suns illumination, the film deposited at 50 Pa outperformed the one deposited at 100 Pa (η_th_ ∼ 35% at *T*_max_ ∼ 462 °C vs η_th_ ∼ 22% at *T*_max_ ∼ 475 °C) because of its lower absorptance in the NIR–MIR range (Figure 4b) and, therefore, lower emittance (Figure 5c). The performance of the hierarchical TiN_*x*_O_*y*_ films at moderate temperature conditions is therefore lower than that of spectrally selective solar absorbers,^23,64^ which is not surprising since this work did not address a control of the spectral emissivity of the TiN/TiN_*x*_O_*y*_ films. However, compared to broadband absorbers made of complex TiN metamaterial structures,^7,69^ the hierarchical nanoporous TiO_*x*_N_*y*_ films could represent a potential alternative thanks to their relatively simple preparation process. In fact, the present results could be exploited to develop more advanced films limiting their emissivity in the MIR range. For example, compact/porous multilayer structures could be realized in the same PLD process,^23,70^ or antireflection coatings could further be included.^66,67^ More complex architectures, such as nanopatterned surfaces,^71^ could also be designed thanks to the possibility of depositing TiN_*x*_O_*y*_ at room temperature on plastic substrates.

## Conclusions

Titanium (oxy)nitride films of tunable morphology, structure and composition were deposited by pulsed laser deposition at room temperature. By increasing the deposition background pressure from vacuum to 100 Pa, the film properties changed from bulk-like TiN_*x*_ to hierarchical nanoparticle assembly of TiO_*x*_N_*y*_. In particular, the hierarchical nanoporous films exhibited an ultrafine nanocrystalline structure with a high degree of oxygen incorporation promoted by the high background gas (N_2_/H_2_) pressure during deposition. The light absorption of the films increased with the deposition pressure, thus allowing a broadband absorptance from the UV to IR wavelength range. The films were studied as perspective candidates for solar–thermal applications by measuring the temperature produced under solar-simulated irradiation with moderate light concentration, which revealed the superior performance of the porous TiO_*x*_N_*y*_ films (maximum temperature ∼475 °C under 17 Suns) and resistance to oxidation. Further performance optimization could be addressed by simple design strategies thanks to the flexibility of pulsed laser deposition, such as by realizing compact/porous multilayers or by including antireflection oxide layers.

## Supporting Information Available

EDX microanalysis of the TiN target; high-resolution XPS spectra for the film deposited at 100 Pa; detailed optical spectroscopy results; ellipsometry measurements; details on the thermal camera measurements; top-view SEM images of an uncoated Ti substrate and of all of the investigated films on Ti substrates; temperature profiles under solar irradiation; Raman spectra before and after solar irradiation for all of the investigated films; quantitative chemical analysis by XPS; details on XPS peak fitting for the film deposited at 100 Pa; details on Raman spectroscopy experiments; details on optical spectroscopy experiments; and details on ellipsometry measurements (PDF)
