# Supplementary material for: Nanoporous Titanium (Oxy)nitride Films as Broadband Solar Absorbers
Source: ACS Appl Mater Interfaces. 2022 Apr 18;14(16):18453–63. doi: 10.1021/acsami.2c01185 (PMC9052191; doi:10.1021/acsami.2c01185)
Supplement: Supplementary file 1 — am2c01185_si_001.pdf [file am2c01185_si_001.pdf]

Supporting Information

Nanoporous titanium (oxy)nitride films as broadband solar absorbers

Beatrice R. Bricchi,[a,†] Luca Mascaretti,[b,†] Simona Garattoni,[a] Matteo Mazza,[a] Matteo Ghidelli,[c] Alberto Naldoni,[b,*] Andrea Li Bassi[a,d,*]

[a]Micro- and Nanostructured Materials Laboratory, Department of Energy, Politecnico di Milano, Via Ponzio 34/3, 20133 Milano, Italy

[b]Czech Advanced Technology and Research Institute, Regional Centre of Advanced Technologies and Materials, Palacký University Olomouc, Šlechtitelů 27, 77900 Olomouc, Czech Republic

[c]Laboratoire des Sciences des Procédés et des Matériaux (LSPM), CNRS, Université Sorbonne Paris Nord, 93430 Villetaneuse, France

[d]Center for Nanoscience and Technology – IIT@PoliMi, Via Giovanni Pascoli 70/3, 20133 Milano, Italy

[†] These authors contributed equally to this work.

Corresponding authors

[*]andrea.libassi@polimi.it

[*]alberto.naldoni@upol.cz

This file includes:

Supplemental Figures

Figure S1. EDX microanalysis of the TiN target.
Figure S2. High-resolution XPS spectra for the film deposited at 100 Pa.
Figure S3. Detailed optical spectroscopy results.
Figure S4. Ellipsometry measurements.
Figure S5. Details on the thermal camera measurements.
Figure S6. Top view SEM images of an uncoated Ti substrate and of all the investigated films on Ti substrates.
Figure S7. Temperature profiles under solar irradiation.
Figure S8. Raman spectra before and after solar irradiation for all the investigated films.

Supplemental Tables

Table S1. Quantitative chemical analysis by XPS.
Table S2. Details on XPS peak fitting for the film deposited at 100 Pa.

Supplemental Notes

Note S1. Details on the interpretation of Raman spectra.
Note S2. Details on optical spectroscopy measurements.
Note S3. Details on ellipsometry measurements.

Supplemental References

Supplemental Figures

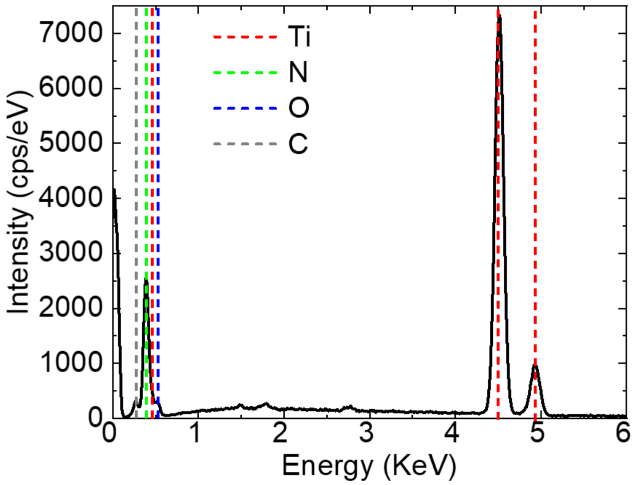

Figure S1. EDX microanalysis of the TiN target. The quantitative reliability of EDX measurements is affected by the small atomic number of nitrogen and oxygen and the vicinity in energy of their x-ray transitions with the lower one of titanium (i.e., $K = 0.392$ keV and 0.525 keV for N and O atoms, respectively; $K = 4.512$ keV and $L = 0.452$ keV for Ti atom). To account for such effects and to ensure reliability of the atomic composition in the films, the nitrogen to titanium ratio of the stoichiometric TiN target was measured and the value N/Ti = 0.8 was found. Moreover, the target also exhibited the presence of oxygen (13% at.), associated to a thin native oxide overlayer which forms on TiN surface because of air exposure.[1]

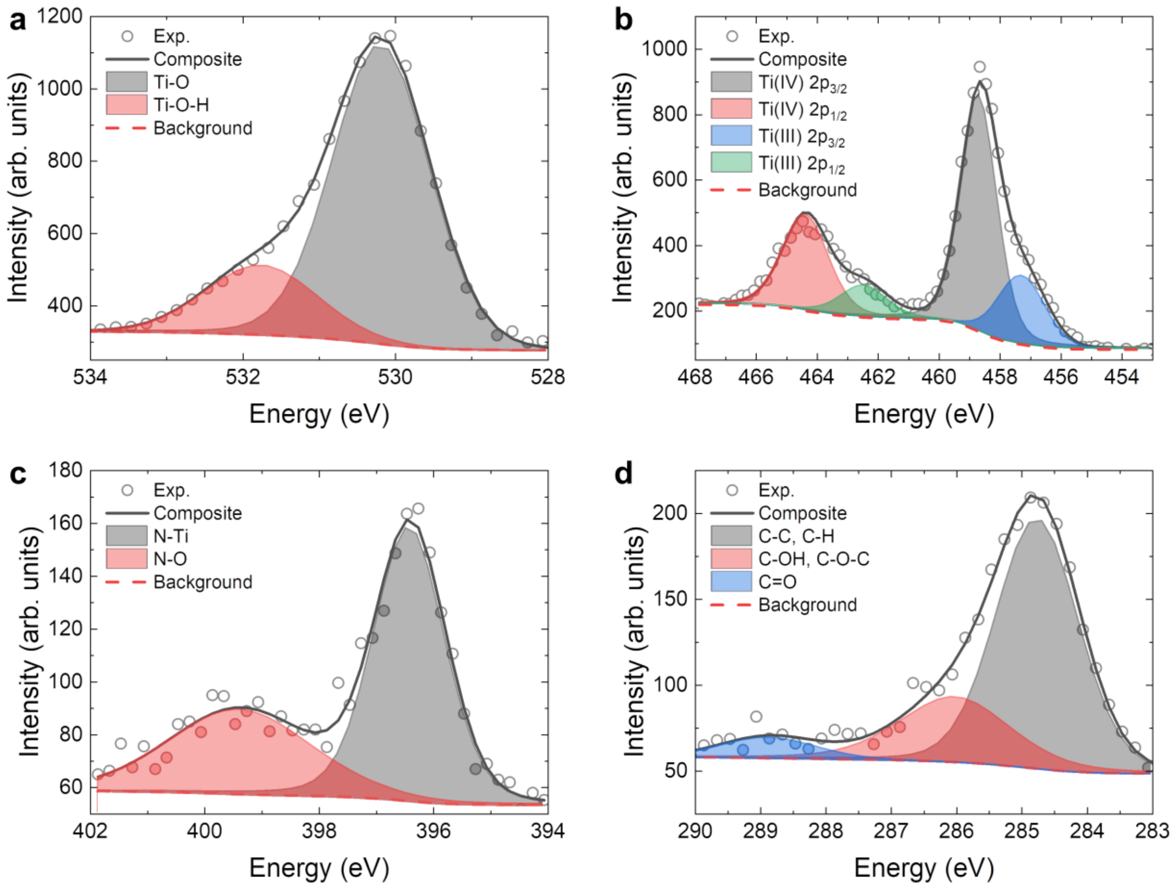

Figure S2. High-resolution XPS spectra for the film deposited at 100 Pa: (a) O 1s, (b) Ti 2p, (c) N 1s, and (d) C 1s regions. The spectra suggest a high oxidation degree of the surface, consistent with a TiO_xN_y material (see also Table S2). The relatively high amount of adventitious carbon (d) can be ascribed to the high porosity of the film.

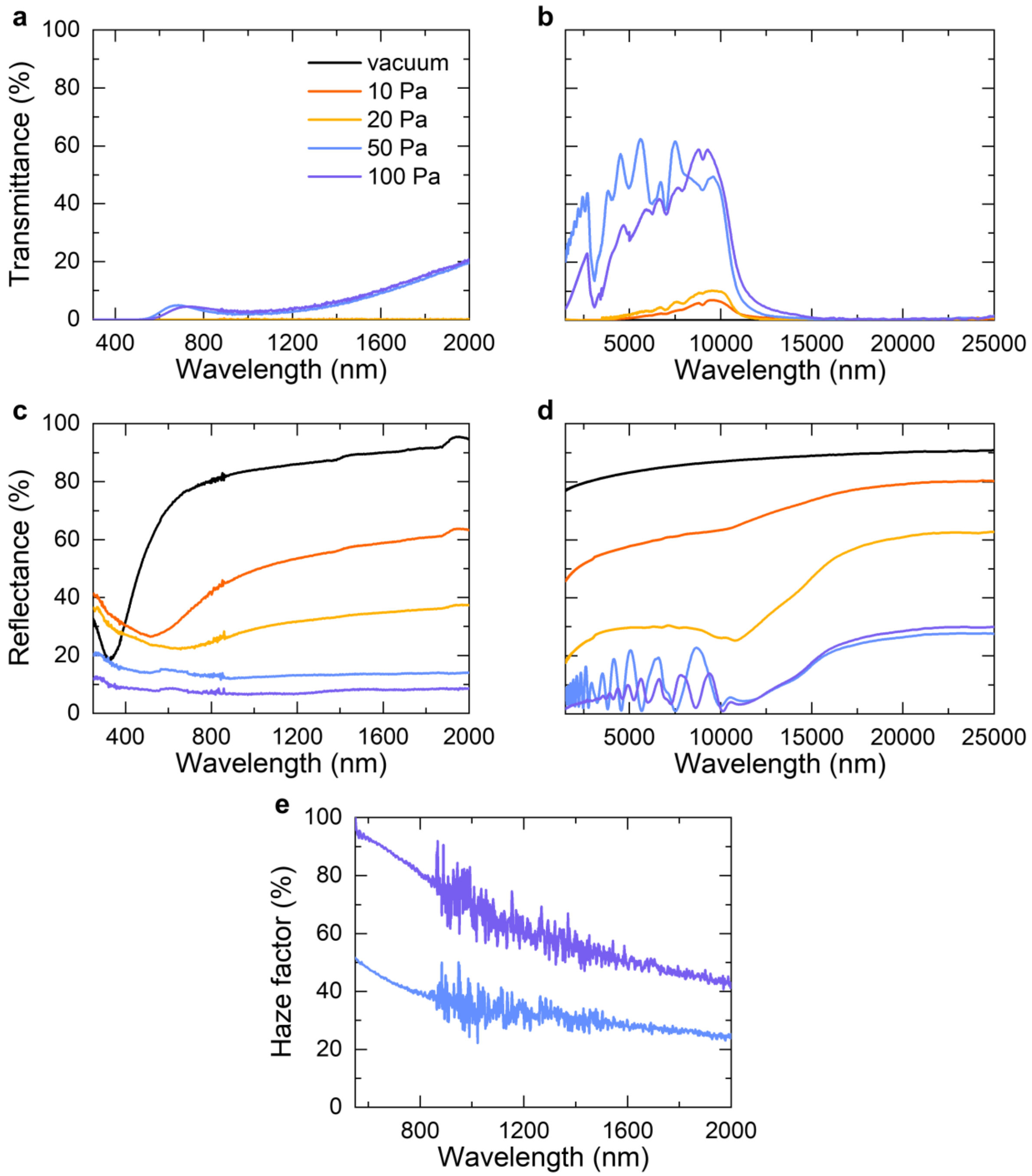

Figure S3. Detailed optical spectroscopy results. (a) Transmittance in the UV-visible-near infrared and (b) in the medium-infrared ranges. (c) Reflectance in the UV-visible-near infrared and (d) in the medium-infrared ranges. (e) Haze factor in the visible-near infrared ranges. For all panels, the color legend is reported in (a). See Note S2 for further details on optical spectroscopy measurements.

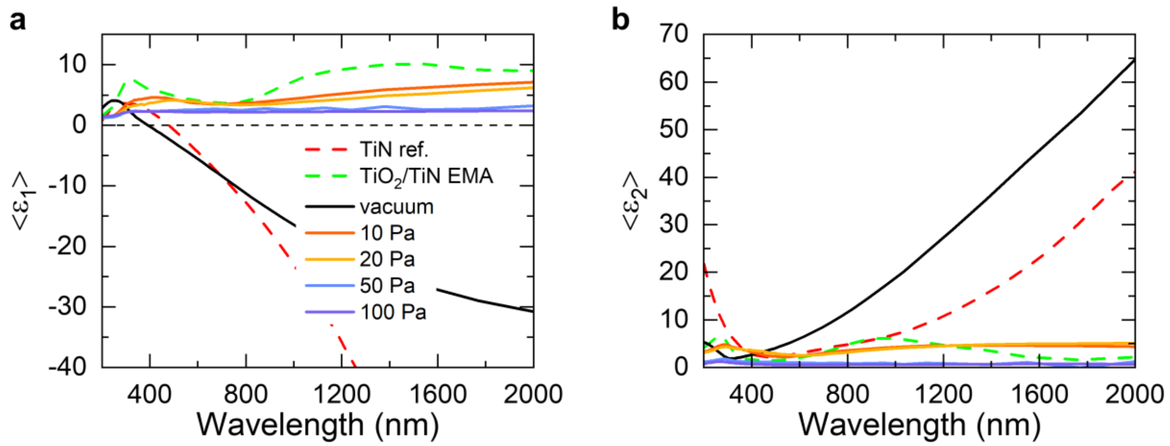

Figure S4. Ellipsometry measurements. Real (a) and imaginary parts (b) of the pseudo-dielectric constants of the TiN_xO_y films obtained by direct inversion of the ellipsometric measurements compared to reference data for TiN (ref. [2]) and for a TiO_2/TiN 75/25% effective medium approximation (EMA) layer (Maxwell-Garnett formalism with TiN inclusions in a TiO_2 matrix). The films deposited in vacuum exhibited a metallic behavior with the crossover wavelength λ_{ps} = 394 nm, i.e., consistent with sub-stoichiometric TiN_x ($x < 1$).[3] The pseudo-dielectric constants of all the other films are, instead, qualitatively consistent with an EMA layer, thus suggesting an extensive oxide fraction with variable TiN_xO_y composition depending on the deposition pressure (see also Figures 2 and 3 in the main text and Table S1). Further details on the ellipsometry measurements are reported in Note S3. It should be noted that the results for the films deposited at 50 and 100 Pa were affected by their low reflectance (Figure S3c) and high haze factor (Figure S3e), which limits the signal collection by the detector in the ellipsometer (which works in specular reflection mode with high angles of incidence, see Note S3).

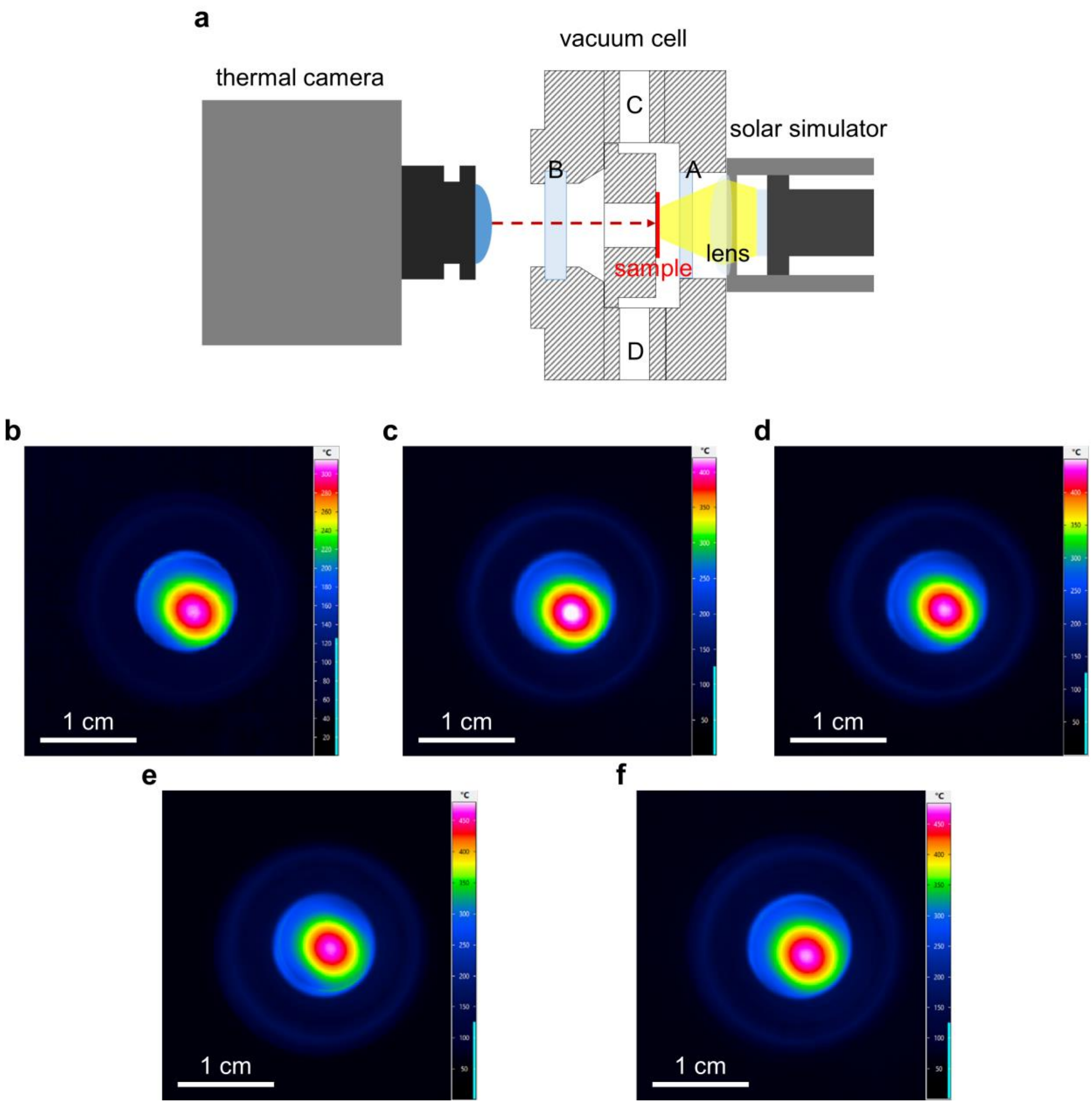

Figure S5. Details on the thermal camera measurements. (a) Schematic of the experimental setup for temperature measurements, in which the custom-made vacuum cell was equipped with a front sapphire viewport (A) and back CaF_2 viewport (B) to allow light transmission for the incident irradiation and the IR thermal camera, respectively, and inlet and outlet gas channels (C and D) to purge air with Ar gas. (b–f) Experimental IR camera images showing the thermal gradients at the back surface of the samples (i.e., from the Ti substrate side) deposited in vacuum (b) and at 10 (c), 20 (d), 50 (e), and 100 Pa (f) under 17 Suns irradiation.

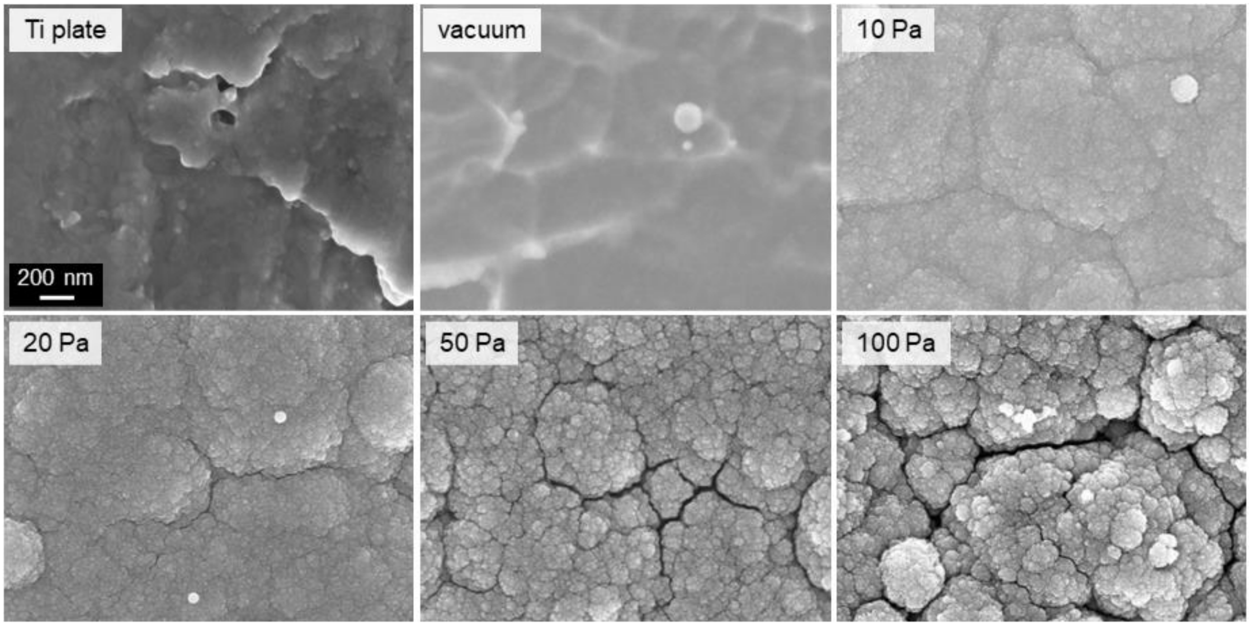

Figure S6. Top view SEM images of an uncoated Ti substrate and of all the investigated films on Ti substrates. These substrates were employed for thermal analysis because they do not affect the morphology of the growing films (see Figure 1 for comparison) but they allowed efficient thermal conduction from the film to the back surface (thickness of the Ti substrate ~ 125 μm).

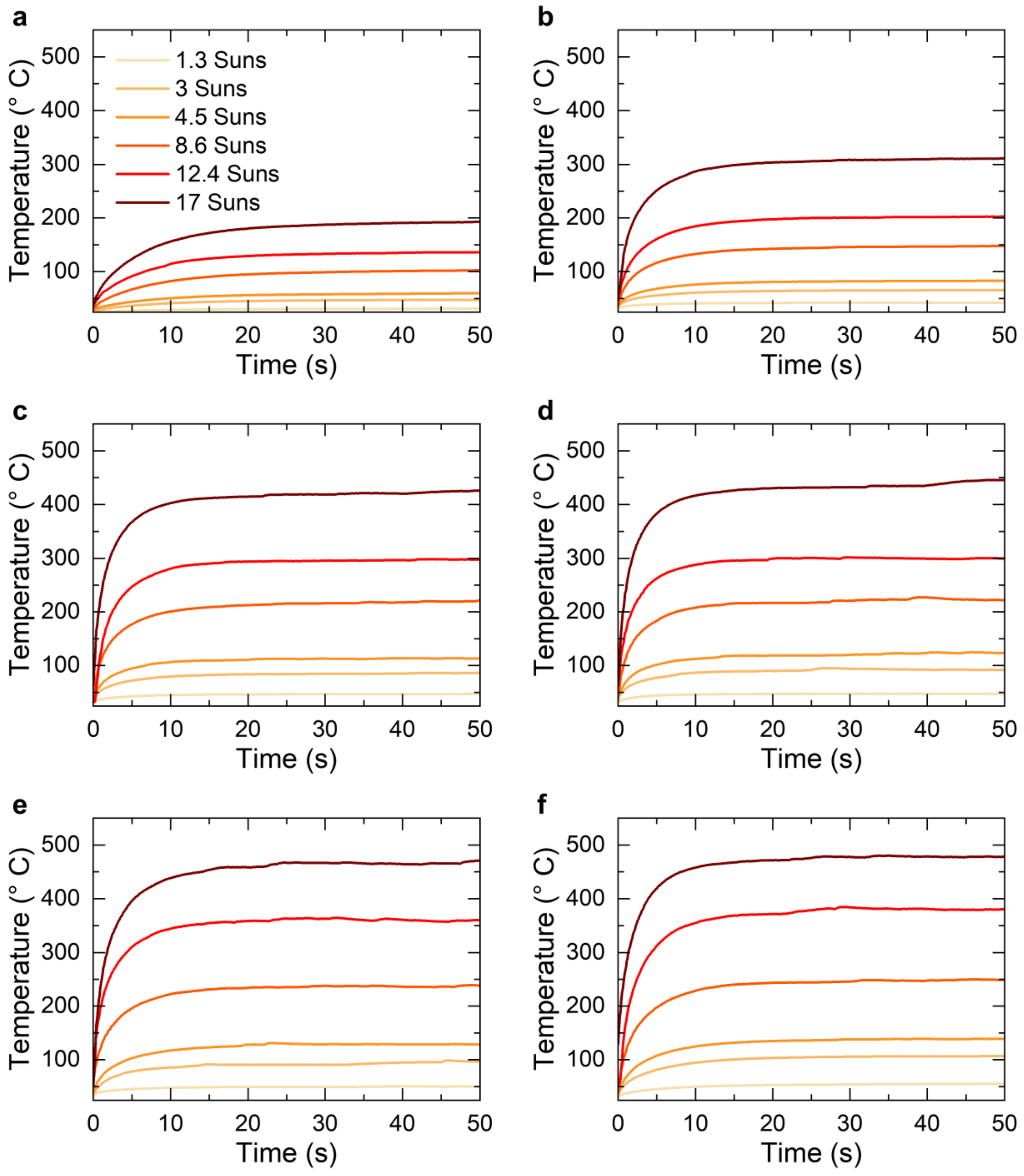

Figure S7. Temperature profiles under solar irradiation. Generated temperature as a function of time at different light intensity (from 1.3 to 17 Suns) for a bare titanium substrate (a) and for the films deposited (b) in vacuum, (c) at 10 Pa, (d) at 20 Pa, (e) at 50 Pa, and (f) at 100 Pa. The color legend for all the panels is reported in (a).

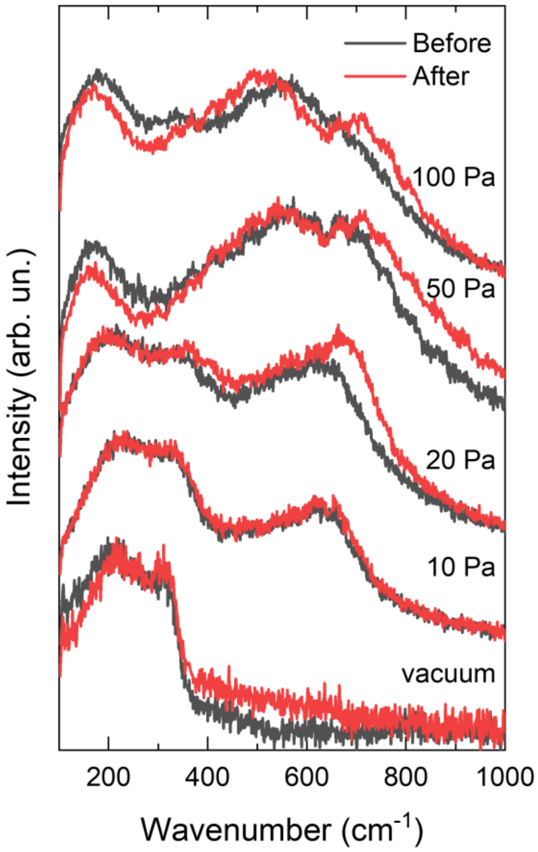

Figure S8. Raman spectra before and after solar irradiation for all the investigated films. Raman spectra after irradiation were acquired on films that were tested for temperature measurements from 1.3 to 17 Suns (Figure S7).

Supplemental Tables

Table S1. Quantitative chemical analysis by XPS. Atomic percentage of Ti, N and O extracted from XPS spectra after 60 s depth profiling and corresponding *N/Ti* ratios. These data were used to plot Figures 3a and 3b.

**P (Pa)**	**Ti (at. %)**	**N (at. %)**	**O (at. %)**	***N/Ti***
vacuum	37.1	26.3	33.3	0.71
10	32.6	22.2	41.0	0.68
20	32.3	27.1	33.3	0.84
50	27.4	13.5	47.0	0.49
100	27.5	13.5	40.4	0.49

Table S2. Details on XPS peak fitting for the film deposited at 100 Pa (Figure S2). A small trace of Si was also detected (1.06% at.) that can be ascribed to the substrate. The assignment of the peaks was based on refs. [4–6] and overall it pointed out a strongly oxidized surface.

**Region**	**% at.**	**Band**	**Pos (eV)**	**Sep (eV)**	**FWHM (eV)**	**% Area**	**χ^2**
O 1s	39.49	Ti–O	530.17		1.51	78.81	2.00
		Ti–O–H	531.75	1.58	1.74	21.19	
Ti 2p	20.48	Ti(III) $2p_{3/2}$	457.25		1.80	16.34	3.32
		Ti(IV) $2p_{3/2}$	458.67	1.42	1.45	50.33	
		Ti(III) $2p_{1/2}$	462.45	5.20	1.80	8.17	
		Ti(IV) $2p_{1/2}$	464.37	7.12	1.80	25.16	
N 1s	10.50	N–Ti	396.40		1.48	61.54	1.09
		N–O	399.39	2.99	3.00	38.46	
C 1s	28.47	C–C, C–H	284.74		1.50	67.68	1.09
		C–OH, C–O–C	286.01	1.27	2.00	25.75	
		O–C=O	288.88	4.13	1.73	6.57	

Supplemental Notes

Note S1. Details on the interpretation of Raman spectra.

In principle, first-order Raman scattering is not allowed in an ideal crystal with rock-salt cubic structure like TiN (i.e., O_h symmetry). However, films deposited by means of physical vapor deposition present defects that may be generated from energetic ions or species in deposition mechanism, leading to a reduction of crystal symmetry which induces first-order Raman modes composed by broad bands. In particular, the relative intensity of acoustic and optical bands can be used as an indication of film stoichiometry. Indeed, acoustic bands occurring at ~ 200–215 cm^{-1} and ~ 300–330 cm^{-1} are associated to vibrations of Ti^{4+} ions due to nitrogen vacancies, while the optical mode at 500–600 cm^{-1} is associated to Ti vacancies and vibration of N^{3-} ions.[7] Therefore, a high $I_{acoustic}/I_{optical}$ ratio (>1) is related to a high number of nitrogen vacancies and, therefore, to under-stoichiometric TiN_x (with $x < 1$), $I_{acoustic}/I_{optical}$ ~1 is associated to nearly-stoichiometric TiN, while $I_{acoustic}/I_{optical}$ <1 over-stoichiometric TiN_x (with $x > 1$).[8]

Note S2. Details on optical spectroscopy measurements.

Optical spectroscopy measurements in the ultraviolet-visible-near infrared (UV-vis-NIR) range were performed in transmittance and reflectance mode on samples grown on glass substrates. The absorptance was calculated according to the formula

$$A(\lambda) = 1 - T(\lambda) - R(\lambda) \tag{S1}$$

where

$$T(\lambda) = T_{spec}(\lambda) + T_{diff}(\lambda) \tag{S2}$$

is the total transmittance, given by the sum of the specular and the diffuse components, and

$$R(\lambda) = R_{spec}(\lambda) + R_{diff}(\lambda) \tag{S3}$$

is the total reflectance, also given as the sum between the specular and diffuse components. The haze factor, on the other hand, was evaluated according to the formula[9]

$$H(\lambda) = T_{diff}(\lambda) / T(\lambda) \tag{S4}$$

The measurement of the total components for both the transmittance and the reflectance was ensured by the use of an integrating sphere in the UV-vis-NIR spectrophotometer. Therefore, Figures S3a, S3c and S3e show the total transmittance, total reflectance and the haze factor, respectively, while Figure 5a in the main text shows the absorptance calculated according to Equation (S1).

Optical spectroscopy measurements in the medium-infrared (MIR) range were instead performed in transmittance and reflectance mode on samples grown on silicon substrates with a FTIR spectrometer, using a thick gold film as a calibration reference for the reflectance and pumping the chamber to vacuum prior to the measurements. The FTIR spectrometer was not equipped with an integrating sphere; therefore, Figures S3b and S3d show the specular transmittance and reflectance, respectively. As a consequence, Figure 5c in the main text shows the absorptance calculated as

$$A_{FTIR}(\lambda) = 1 - T_{spec,FTIR}(\lambda) - R_{spec,FTIR}(\lambda) \tag{S5}$$

This instrumental limitation likely gave rise to a slight difference in absorptance values measured by the two different techniques (i.e., UV-vis-NIR spectroscopy and FTIR spectroscopy) in the NIR region of the electromagnetic spectrum (see Figures 5a and 5c in the main text). Therefore, the absorptance measured by FTIR spectroscopy is overestimated. This effect is more relevant for the porous samples because they exhibited a high light scattering property, i.e., a high haze factor (Figure S3e). Finally, the absorptance was converted to spectral emissivity according to the Kirchhoff's law of thermal radiation, which asserts the equality of absorptivity and emissivity for a body at thermal equilibrium:

$$A_{FTIR}(\lambda, T_0) = \varepsilon_\lambda(\lambda, T_0) \qquad \text{(S6)}$$

Since FTIR spectroscopy measurements could be performed only at room temperature, the thermal dependence of the emissivity could not be considered, which introduces an additional but systematic error in the emissivity values presented in this work.

Note S3. Details on ellipsometry measurements.

Ellipsometric measurements were performed on all the films grown on Si substrates with the angles of incidence φ = 65° and 75° in the energy range 0.6–6.5 eV (0.1 eV interval). The pseudo-dielectric constants were extracted from the experimental data by direct inversion (using the WVASE32 software, J. A. Woollam Co.) according to the formula

$$\langle \tilde{\varepsilon} \rangle = \sin^2\phi \left[1 + \tan^2\phi \left(\frac{1-\rho}{1+\rho} \right) \right] = \langle \varepsilon_1 \rangle + i \langle \varepsilon_2 \rangle \qquad \text{(S7)}$$

where $\rho = R_p / R_s = \tan\Psi e^{i\Delta}$ is the ratio between the parallel (p) and the perpendicular (s) Fresnel reflection coefficients and Ψ and Δ are the so-called ellipsometric angles.[10] Such nomenclature is used because the dielectric constants obtained by direct inversion represent an overall behavior of the material, thus including the effects of roughness and multi-layers. The actual permittivity of the constituent materials may be obtained by fitting the experimental data by appropriate oscillators depending on the nature of the materials, i.e., Drude-Lorentz model for metallic materials, Tauc-Lorentz model for oxides, etc.

Supplemental References

(1) Ernsberger, C.; Nickerson, J.; Smith, T.; Miller, A. E.; Banks, D. Low Temperature Oxidation Behavior of Reactively Sputtered TiN by X-ray Photoelectron Spectroscopy and Contact Resistance Measurements. *Journal of Vacuum Science & Technology A* **1986**, *4* (6), 2784–2788. https://doi.org/10.1116/1.573679.

(2) Reddy, H.; Guler, U.; Kudyshev, Z.; Kildishev, A. V.; Shalaev, V. M.; Boltasseva, A. Temperature-Dependent Optical Properties of Plasmonic Titanium Nitride Thin Films. *ACS Photonics* **2017**, *4* (6), 1413–1420. https://doi.org/10.1021/acsphotonics.7b00127.

(3) Kang, J. H.; Kim, K. J. Structural, Optical, and Electronic Properties of Cubic TiNx Compounds. *J. Appl. Phys.* **1999**, *86* (1), 346–350. https://doi.org/10.1063/1.370736.

(4) Saha, N. C.; Tompkins, H. G. Titanium Nitride Oxidation Chemistry: An X-ray Photoelectron Spectroscopy Study. *J. Appl. Phys.* **1992**, *72* (7), 3072–3079. https://doi.org/10.1063/1.351465.

(5) Biesinger, M. C.; Lau, L. W. M.; Gerson, A. R.; Smart, R. St. C. Resolving Surface Chemical States in XPS Analysis of First Row Transition Metals, Oxides and Hydroxides: Sc, Ti, V, Cu and Zn. *Appl. Surf. Sci.* **2010**, *257* (3), 887–898. https://doi.org/10.1016/j.apsusc.2010.07.086.

(6) Jaeger, D.; Patscheider, J. A Complete and Self-Consistent Evaluation of XPS Spectra of TiN. *J. Electron Spectrosc. Relat. Phenom.* **2012**, *185* (11), 523–534. https://doi.org/10.1016/j.elspec.2012.10.011.

(7) Spengler, W.; Kaiser, R. First and Second Order Raman Scattering in Transition Metal Compounds. *Solid State Communications* **1976**, *18* (7), 881–884. https://doi.org/10.1016/0038-1098(76)90228-3.

(8) Rasic, D.; Sachan, R.; Chisholm, M. F.; Prater, J.; Narayan, J. Room Temperature Growth of Epitaxial Titanium Nitride Films by Pulsed Laser Deposition. *Crystal Growth & Design* **2017**, *17* (12), 6634–6640. https://doi.org/10.1021/acs.cgd.7b01278.

(9) Dominé, D.; Haug, F.-J.; Battaglia, C.; Ballif, C. Modeling of Light Scattering from Micro- and Nanotextured Surfaces. *J. Appl. Phys.* **2010**, *107* (4), 044504. https://doi.org/10.1063/1.3295902.

(10) Tompkins, H. G.; Hilfiker, J. N. *Spectroscopic Ellipsometry: Practical Application to Thin Film Characterization*; Momentum Press, 2016.
